# Progress in Surgery

**Published:** 1895-11-16

**Authors:** 


### Progress in Surgery.

### GENITO-URINARY SURGERY.

**Prostatic Hypertrophy.**—Since the account given in The Hospital on March 30th of the treatment of prostatic obstruction by bilateral castration, numerous cases have been operated on with success. Dr. J. William White, of Philadelphia, has collected the records of 111 cases and published them[1] in tabular form, with a careful and thorough review of the whole subject. Dr. White considers that the theoretical objections which have been urged against the operation of double castration have been fully negatived by clinical experience, which shows that in about 87 per cent. rapid atrophy of the prostatic enlargement follows the operation, and that cystitis disappears or greatly improves with more or less return of vesical contractility, and in a considerable number of cases a return to local conditions not very far from normal may be expected. In the 111 cases there were twenty deaths, or a mortality of 18 per cent., but Dr. White thinks thirteen of these may be excluded in an attempt to ascertain the legitimate mortality in patients operated on under surgically favourable conditions, for they were desperate cases with anæmia or great exhaustion, and the patients would probably have died without any operation. Fauld's (Glasgow) cases form such a remarkable series that they can hardly be estimated as influencing the mortality statistics. Dr. White considers 7·1 per cent. the mortality at present for cases not practically in a dying condition before the operation is undertaken. The interesting and important question is discussed as to whether unilateral removal or atrophy of the testis causes atrophy of the prostate on the same side. There is very clear evidence that in cases of retained and atrophied testis the prostate is sometimes found also atrophied on the same side, and in some cases of atrophy of the testis from other causes, or its removal, the prostate has been found atrophied on the same side, but by no means invariably so. In two cases of prostatic hypertrophy in which one testicle was removed for other conditions, distinct improvement occurred in the symptoms of prostatic obstruction, with diminution in the size of the gland chiefly on the side of the castration.[3] In one case unilateral castration was performed for the relief of the prostatic obstruction with good result.[4] As mentioned in the "Surgical Progress," March 30th, Mr. Reginald Harrison, in one case, was asked by the patient (a medical man) to perform castration for prostatic hypertrophy, but instead divided both vasa-deferentia with doubtful result. Griffiths has lately carried out a series of experiments to determine the effect on the testis of ligation of the vas deferens.[5] He finds that not only does the occlusion of the vas fail to produce atrophy of the fully-developed testis in dogs, but it does not interfere with its development in puppies. Dr. White has just been conducting a series of experiments on dogs to ascertain the effect on the prostate of ligation of the vas, and he found a constant loss of weight in the prostate in every dog that died after eight days. The effects on the testes were the same as in Griffiths' experiments, hence atrophy of testis was not the cause of the prostatic atrophy. Dr. White cannot help thinking he may have included a few fine nerves with the vas, and that their inclusion caused the prostatic atrophy. He also found that ligation of the vascular constituents of the cord, or of the whole cord, produced atrophy of the prostate, but only after causing sloughing of the testes. The question is discussed, When should castration be resorted to in cases of prostatic enlargement? Dr. White does not think that it is called for so long as residual urine can be drawn off, and there is no cystitis. He lays down the rule that in such patients, with but moderate obstruction, or with a high grade of compensatory hypertrophy of the bladder, with a small amount of residual urine which remains sterile, and in whom catheterisation is easy and painless, operation is not to be thought of. On the other hand, operation may be undertaken with hope of success even if the urine has only been passed by catheter for a long time, as Dr. White considers Sir Henry Thompson's assertion that the return of voluntary power in the bladder is impossible after two years' use of the catheter, is incorrect. Dr. White himself records the following case:[6] Patient æt. 60, had had symptcms of prostate obstruction for 18 months, was urinating hourly, and had used the catheter with increasing frequency for several years, and was in great distress. The urine was stinking, and contained blood and muco-pus. The prostate was as large as a small orange, and there was an average of six ounces of residual urine. A week after bilateral castration the patient emptied the bladder easily and painlessly, the urine was clear, and the frequency of micturition much diminished, and the amount of residual urine had diminished to half a fluid ounce. The prostate atrophied to such an extent that it could hardly be felt.

Pavone[7] also has made a series of experiments on dogs to ascertain if excision of the vas-deferens will produce prostatic atrophy, and he finds that it does so, thus confirming the results of White's experiments. Microscopic examination of the prostate after excision of both vasa-deferentia shows that the same atrophic change takes place as after removal of the testes. Kummell[8] records eight cases of castration from prostatic hypertrophy not included in White's series of 111 cases. The operation was followed by considerable relief, &c., the patients were able to dispense with the aid of the catheter, and to pass urine spontaneously. The author met with no objection to the operation from the patients, and they were all well satisfied with the results. Bryson[9] reports a case in which the operation was quite painlessly carried out after the injection of cocaine along the line of incision and into the substance of each cord, after an elastic ligature had been applied around the root of the scrotum. A little more than a drachm of a 4 per cent. solution was used. No ill effects followed, although the dose was so large. The patient was 74 years of age. Mr. Henry Fenwick[10] thinks that castration is attended by a more severe degree of shock than other operations of a similar gravity and duration, and that it may, together with the anæsthesia, cause an upset in the mental equilibrium of elderly patients with prostatic obstruction, in whom the mental equilibrium is often very unstable. Mr. Fenwick also points out that if the enlargement of the prostate is in the adenomatous stage recovery is rapid; but if fibroid induration has set in, it may be some months before there is any definite improvement.

**Tuberculous Disease of the Bladder.**—Until the last few years surgery offered little in the way of treatment for tuberculous disease of the bladder, but lately several cases have been reported in which marked improvement or cure have followed suprapubic drainage of the bladder, and the partial destruction of the ulcerated area in the bladder by cauterisation or scraping. Mr. Watson Cheyne reports four such cases.[11] They were all in males, and, with one exception, the disease was confined to the bladder, and the ulcers were treated by scraping and the application of iodoform at the time of operation. Mr. Watson Cheyne considers that the good results obtained were due to the rest obtained for the bladder, and does not think the scraping could be thorough enough in the lax bladder wall to eradicate the tuberculous material, or that one application of iodoform can do any appreciable good. He drains until he thinks the tubercular disease has come to an end—for many weeks or months. Tubercle bacilli were discovered in the urine in two of the cases. In the first case the symptoms were very acute, the patient passing blood at the end of micturition. Patches of intense congestion, with slight ulceration on their surface, were found in the trigone, and several miliary tubercles were seen in the mucous membrane near them. He completely recovered. In the second case there were the usual symptoms, and an ulcer was discovered by the cystoscope in the trigone, and a hard nodule felt per rectum in the base of the bladder. Drainage had to be continued for six months; but by that time the nodule had disappeared and he had none of the old symptoms after drainage was discontinued. The third case was one in which, although the symptoms were of long duration, no actual ulceration was found in the bladder, but suppurative tuberculous disease subsequently developed in one kidney necessitating nephrectomy. After prolonged drainage of the bladder the boy recovered. The fourth case was that of a man forty-nine years of age, and the lesion discovered was a thickened mass in the anterior wall of the bladder from which a quantity of cheesy material was scraped away. No iodoform was used, and the bladder was drained for six weeks. He quickly recovered. Mansell Moullin[12] also records a case of tuberculous disease of the bladder treated by suprapubic cystotomy and cauterisation of the ulcers. The patient was a young woman aged twenty-two, and the symptoms of vesical irritability were intense, the urine containing much pus, blood, and phosphates, and once tubercle bacilli were found in it. After several months' treatment by means of irrigation of the bladder without improvement, several ulcers, coated over with an adherent, bloodstained, caseous deposit, were found just inside the orifice. When the bladder was opened more ulcers were discovered on the anterior wall; those at the base were cauterised, and those on the anterior wall scraped and iodoform rubbed in. All the ulcers were situated near the orifice of the bladder, there were none on the fundus; drainage in this case was only continued for a few days, and the bladder wound was healed in three weeks. Recovery was satisfactory. Mansell Moullin gives references to the previously recorded cases of cystotomy for tubercular disease of the bladder (which are about a dozen), and states that, in nearly every case of cauterisation, the results as reported were good; and he thinks there is little doubt that, in many instances, the operation might be resorted to with advantage at a much earlier period than it usually is. Moullin considers that ulceration of the bladder, attended by stranguary and hæmaturia, is of not uncommon occurrence in young adults of both sexes, and in a large proportion of cases yields readily to local palliative treatment; and in such cases the ulceration is not tuberculous, or if so, it is only very superficial. When the ulcers are deep and coated over with caseous débris, and pus, and phosphates, they can only be satisfactorily dealt with by suprapubic cystotomy; and, seeing that the prognosis is so unfavourable and the suffering so intense, and the risks of suprapubic cystotomy so slight, Moullin thinks it ought to be oftener resorted to. He considers there is rather a tendency to exaggerate the frequency of tuberculous disease of the bladder being usually secondary to similar disease elsewhere in the genito-urinary track; and thinks that, as many of the cases which have occurred have been in women who do not suffer in the same way from genital tuberculosis, and whose kidneys were healthy, many of the cases which occur in males may also be primarily vesical. Dr. Bolton Bangs, of New York, advocates[13] hygienic and general treatment in the early stage of the disease, with which all surgeons will agree; but the cases just referred to show that there is more hope for patients in whom the disease is far advanced from operative interference than Dr. Bangs seems to think. Watson[14] considers that suprapubic cystotomy for relief of vesical tenesmus is markedly successful, especially when supplemented by applications, such as curetting and the application of iodoform to the ulcers.

**Rupture of the Bladder.**—Mr. Walsham reports a case[15] in which the test of inflation with air was tried, and proved very successful as a diagnostic measure. The air was forced in by means of two or three compressions of the rubber ball of an ether-freezing microtome. The abdomen at once became tympanitic, and the liver dulness effaced. Mr. Walsham, however, considered that the entrance of air into the peritoneal cavity caused serious collapse, and suggests that it should not be undertaken until the patient is on the operating table, and the abdomen can be at once opened to let out the air. Milk was injected into the bladder to test the efficiency of the sutures. The patient made an uninterrupted recovery. The conclusions drawn from the experience of this case—the first in which the air test has been employed—were: (1) That the amount of air to be introduced need only be very small; (2) that only very moderate pressure was required for the inflation; (3) that the presence of quite a small amount of free gas in the abdominal cavity was sufficient to establish the diagnosis without a doubt. Of 28 cases of suture of the bladder for intra-peritoneal rupture, 11 recovered and 17 died. In 4 out of the 28 cases the bladder was found to leak at the post-mortem examination, and the importance of testing the competency of the bladder by injecting milk or other bland fluid could not be too strongly urged. In half the fatal cases peritonitis had already set in at the time of operation; most of the other half died from shock.

**Surgery of the Ureters.**—Büdinger (Vienna) considers[16] that if the ureter be divided during an operation it is not good surgery to form a ureteral fistula in the abdominal wall, for it is always an annoyance to the patient, and the kidney is exposed to the risk of septic infection. The author mentions the following methods of uniting the divided ureter: (*a*) Insertion of a soft catheter into the divided ureter, and its withdrawal before the last sutures are tied; (*b*) opening of the bladder, and then passing a catheter into the ureter, the cut ends being sutured over the catheter, which can then be left in place several days; (*c*) dilatation of the lower segment, and drawing the upper segment into it before suturing; (*d*) tying the lower end, and making a slit in the side through which the upper end can be drawn. The results of implantation of the ureters into the rectum have not been satisfactory, as the ureters have either broken loose or infection has passed up them to the kidneys. The urine can, however, be retained by the rectum without producing any irritation, and the sphincter is strong enough to allow the rectum to act as a reservoir for it. Implantation into the bladder is said to promise good results. An incision is made in the anterior wall of the bladder, and then a small one in the posterior wall, through which the ureter can be drawn and held in place with sutures. Another row of sutures is inserted on the outside of the bladder. The author has improved on this method. Two folds of the bladder wall are drawn by sutures and fixed over the ureter after its insertion into the bladder, thus building a canal in which it runs. No sutures are put into the bladder, as they form the starting point for concretions. It is claimed for this method that there is no leakage of urine after the junction is made, as filling of the bladder tends to obliterate the canal.

Krug (New York) records a successful case of implantation of the ureter, accidentally divided in the removal of a very large uterine fibroid into the bladder. Only one incision near the vertex of the bladder was made, the ureter drawn within the organ with fine catgut sutures passed through its walls, and the ureter then carefully sutured to it from the outside. The bladder was emptied by catheter for a week. In cases of removal of uterine or ovarian tumours, where it is feared one ureter has been accidentally ligated, Kelly (Baltimore) has performed exploratory ureterotomy, and then passed a catheter down the ureter into the bladder to ascertain if it were patent. In one case he discovered by this means that the ureter had been included in his ligature, the ligature was untied, and the catheter passed on. The ureteral wound was, of course, accurately closed with fine sutures. Kelly also records a very successful case of implantation of the ureter into the bladder for uretero-vaginal fistula following vaginal hysterectomy. The ureter was brought forwards after division of the peritoneum, covering it at the brim of the pelvis, and the bladder was separated from the pubes on both sides, and dropped back into the pelvis so as to meet the shortened ureter. The ureter was drawn into the bladder through a small incision by means of long, fine forceps passed through the urethra. A similar case was communicated to the Belgian Academy of Medicine by Rouffart.[19] Roberts records a case of transperitoneal ureterolithotomy. This unusual method of reaching the stone was undertaken under the supposition that the stone was a tumour. Symptoms of renal colic had been marked, and an obstruction in the ureter demonstrated by catheterisation of the ureters from the bladder (the patient was a woman). The kidney and ureter were explored by the lumbar incision, and on passing a long bougie down the ureter an obstruction was discovered at its lower end, where a small swelling could then be detected by vaginal examination. This swelling was supposed to be a tumour pressing on the ureter, but was a small calculus pushed down into this position by the bougie, as Roberts discovered when he opened the abdomen in order to remove it at a subsequent operation. Kelly describes[21] a method by which the orifices of the ureters can be inspected, by means of light reflected through a speculum passed through the dilated urethra. The ureteral orifices can be catheterised, or even the pelvis of the kidney, as Kelly successfully accomplished in one case, evacuating the pus from a pyonephrosis.

A very remarkable case of enormous dilatation of both ureters and the pelvis of each kidney in a newborn infant is recorded by Mr. H. Morris.[22] The dilated ureters formed large abdominal swellings, and were drained by lumbar incisions after an exploratory laparotomy had settled their nature, and the infant lived ninety-four days. The bladder was much hypertrophied, as the cause of the obstruction was an unusual degree of development of the penis with twisting of the organ. The case is a good illustration of the way in which similar changes occur in the urinary organs after obstruction, in fœtal life, as are found to result from obstruction, from stricture, or other causes, after birth.

[1] Annals of Surgery, July, 1895, p. 1. [2] Brit. Med. J., March 9 and 30, April 6, and May 11, 1895. [3] Haynes' Medical Record, May 11, 1895; Langton, Brit. Med. Journal, 1895, Vol. I., p. 979. [4] Haynes' Annal Surgery, July, 1895, p. 41. [5] Lancet, 1895, Vol. VI., p. 917. [6] Annals Surgery, July, 1895, p. 13. [7] Il Policlinico, June 1, 1895, and Epitome B. Med. J., July 6, 1895. [8] Berl. Klinik, Aug., 1895, and Epitome B. Med. J., Aug. 17, 1895. [9] N. York Med. J., April 27, 1895. [10] B, Med. Journal, May 11, 1895, [11] Lancet, June 22, 1895. [12] Lancet, May 25, 1895. [13] Annals Surgery, Aug., 1895, p. 205. [14] Boston Medical and Surgical Journal, Feb. 7, 1895. [15] Lancet, June 15, 1895. [16] Annals of Surgery, Feb., 1895, p. 220. [17] Annals of Surgery, May, 1895, p. 614, [18] Ibid., Sept., 1895, p. 410. [19] Med. Week, June 11, 1895. [20] Annals of Surgery, Sept., 1895, p. 363. [21] Med. News, May 11, 1895, ard Epitome B.M.J., June 22, 1895. [22] Lancet, June 8, 1895, p. 1,435.

